# Trimeth­yl(triphenyl­meth­oxy)silane

**DOI:** 10.1107/S1600536812003170

**Published:** 2012-01-31

**Authors:** Sumati Anthal, Bubun Banerjee, Goutam Brahmacharia, Rajni Kant, Vivek K. Gupta

**Affiliations:** a Post-Graduate Department of Physics and Electronics, University of Jammu, Jammu Tawi 180 006, India; b Laboratory of Natural Products and Organic Synthesis, Department of Chemistry, Visva-Bharati University, Santiniketan 731 235, West Bengal, India.

## Abstract

In the title mol­ecule, C_22_H_24_OSi, the Si—O—C angle is 139.79 (11)°, the O—C—C angles of the triphenyl­meth­oxy group are in the range 106.13 (13)–109.22 (14)° and the O—Si—C angles of the trimethyl­sil­yloxy group are in the range 103.08 (10)–113.53 (10)°. In the crystal, face-to-face π–π interactions are observed between the phenyl rings [centroid separation = 4.194 (1) Å, interplanar spacing = 3.474 Å and centroid shift = 2.35 Å]. The three phenyl groups of the triphenyl­methyl substituent are mutually nearly perpendicular, with dihedral angles in the range 80.49 (8)–81.53 (8)°. There are only weak inter­molecular van der Waals inter­actions in the crystal.

## Related literature

For general background of trimethyl­silylation of alcohols and phenols, see: Kocienski (1994 ▶); Greene & Wuts (1999 ▶).
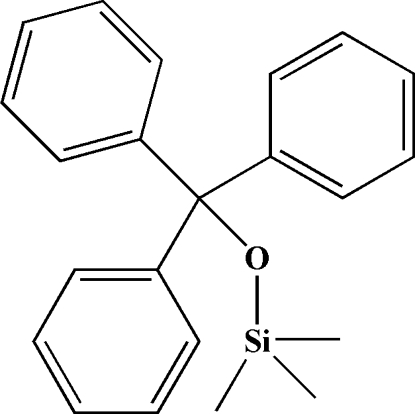


## Experimental

### 

#### Crystal data

C_22_H_24_OSi
*M* *_r_* = 332.50
Triclinic, 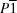

*a* = 9.7621 (6) Å
*b* = 10.6324 (5) Å
*c* = 10.7923 (5) Å
α = 103.412 (4)°
β = 115.389 (5)°
γ = 92.133 (4)°
*V* = 972.41 (9) Å^3^
*Z* = 2
Mo *K*α radiation
μ = 0.13 mm^−1^
*T* = 293 K
0.3 × 0.2 × 0.2 mm

#### Data collection

Oxford Diffraction Xcalibur Sapphire3 diffractometer
Absorption correction: multi-scan (*CrysAlis PRO*; Oxford Diffraction, 2010 ▶) *T*_min_ = 0.876, *T*_max_ = 1.000
10153 measured reflections
3813 independent reflections
2586 reflections with *I* > 2σ(*I*)
*R*_int_ = 0.034

#### Refinement

*R*[*F* ^2^ > 2σ(*F* ^2^)] = 0.049
*wR*(*F* ^2^) = 0.133
*S* = 1.03
3813 reflections
220 parameters
H-atom parameters constrained
Δρ_max_ = 0.21 e Å^−3^
Δρ_min_ = −0.20 e Å^−3^

### 

Data collection: *CrysAlis PRO* (Oxford Diffraction, 2010 ▶); cell refinement: *CrysAlis PRO*; data reduction: *CrysAlis PRO*; program(s) used to solve structure: *SHELXS97* (Sheldrick, 2008 ▶); program(s) used to refine structure: *SHELXL97* (Sheldrick, 2008 ▶); molecular graphics: *ORTEP-3* (Farrugia, 1997 ▶); software used to prepare material for publication: *PLATON* (Spek, 2009 ▶) and *PARST* (Nardelli, 1995 ▶).

## Supplementary Material

Crystal structure: contains datablock(s) I, global. DOI: 10.1107/S1600536812003170/gk2444sup1.cif

Structure factors: contains datablock(s) I. DOI: 10.1107/S1600536812003170/gk2444Isup2.hkl

Supplementary material file. DOI: 10.1107/S1600536812003170/gk2444Isup3.cml

Additional supplementary materials:  crystallographic information; 3D view; checkCIF report

## Figures and Tables

**Fig. 1.**
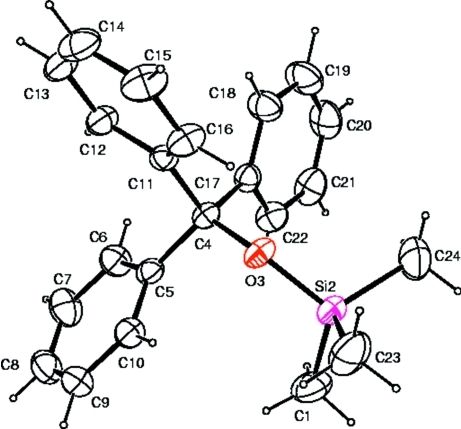
ORTEP view of the molecule with the atom-labeling scheme. The displacement ellipsoids are drawn at the 40% probability level. H atoms are shown as small spheres of arbitrary radii.

**Fig. 2.**
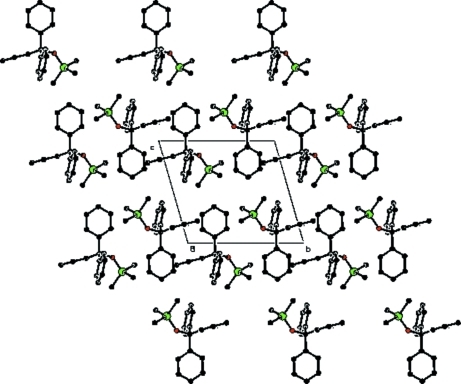
The packing arrangement of molecules viewed down the a axis.

## supplementary crystallographic information

### Comment

Trimethylsilylation of hydroxyl group in organic compounds finds immense applications in preparative organic chemistry (Kocienski, 1994; Greene & Wuts, 1999). Trimethylsilyl is regarded as one of the most popular and widely used groups for protecting hydroxyl function in several chemical conversions and multi-step organic syntheses of poly-functional compounds. Silylethers are also valuable synthetic monomers for the production of organosilane polymers and materials. Several methods were reported for the trimethylsilylation of alcohols and phenols using a variety of silylating agents in the presence of catalysts however most of the reported methods of trimethylsilylation do not work well to furnish 2,2-dimethyl-2-triphenylmethoxy-2-silaethane from triphenylmethanol. Under this purview, we have been motivated to develop an efficient method for the synthesis of the title compound from triphenylmethanol. In continuation of our efforts to develop useful synthetic methodologies for organic transformations, we herein wish to report a newly developed synthetic protocol and crystal structure of 2,2-dimethyl-2-triphenylmethoxy-2-silaethane.

Phenyl ring (C11—C16) makes a dihedral angle of 80.55 (8)° and 81.53 (8)° with the phelyl rings (C5—C10) and (C17—C22), respectively. One of the phenyl rings (C5—C10) in the molecule is involved in face-to-face π-π interaction. The Si—O bond length is 1.6379 (14) Å and Si–O–C bond angle is 139.79 (11)°. Owing to the absence of any strong donor group, cohesion of the crystal is mainly achieved by van der Waals interactions (Fig.2). The closest contact of 3.611 (4) Å occurs between atoms C8 and C10 (-*x* + 2, -*y* + 2, -*z*).

### Experimental

The synthesis of the title compound was carried out following a newly developed methodology. An oven-dried screw cap test tube was charged with a magnetic stir bar, dehydrated copper chloride (0.0067 g, 0.05 mmol), triphenylmethanol (0.26 g, 1 mmol) and trimethylsilyl cyanide (TMSCN, 0.198 g, 2 mmol). The tube was then evacuated and back-filled with nitrogen. The tube was placed in a preheated oil bath at 170°C and the reaction mixture was stirred vigorously for 1 h. The progress of the reaction was monitored by TLC, and on completion the reaction mixture was cooled to room temperature. Dried ethyl acetate (10 ml) was added and shaken well; copper chloride was removed by filtration. The filtrate was concentrated under reduced pressure and the residue was purified *via* column chromatography using silica gel (60–120 mesh) and petroleum ether(PE)-ethyl acetate (EtOAc) (98:2) mixture as eluent. The recrystallization of the solid product from PE/EtOAc afforded the title compound (302 mg, yield 91%) with the m.p. 323–325 K.

### Refinement

All H atoms were positioned geometrically and were treated as riding on their parent C atoms, with C—H distances of 0.93–0.96 Å; and with *U*_iso_(H) = 1.2U_eq_(C), except for the methyl groups where *U*_iso_(H) = 1.5U_eq_(C).

### Crystal data

**Table:**

C_22_H_24_OSi	*Z* = 2
*M**_r_* = 332.50	*F*(000) = 356
Triclinic, *P*1	*D*_x_ = 1.136 Mg m^−^^3^
Hall symbol: -P 1	Mo *K*α radiation, λ = 0.71073 Å
*a* = 9.7621 (6) Å	Cell parameters from 4215 reflections
*b* = 10.6324 (5) Å	θ = 3.8–29.2°
*c* = 10.7923 (5) Å	µ = 0.13 mm^−^^1^
α = 103.412 (4)°	*T* = 293 K
β = 115.389 (5)°	Block-shaped, white
γ = 92.133 (4)°	0.3 × 0.2 × 0.2 mm
*V* = 972.41 (9) Å^3^	

### Data collection

**Table:**

Oxford Diffraction Xcalibur Sapphire3 diffractometer	3813 independent reflections
Radiation source: fine-focus sealed tube	2586 reflections with *I* > 2σ(*I*)
graphite	*R*_int_ = 0.034
Detector resolution: 16.1049 pixels mm^-1^	θ_max_ = 26.0°, θ_min_ = 3.8°
ω scans	*h* = −12→11
Absorption correction: multi-scan (CrysAlis PRO; Oxford Diffraction, 2010)	*k* = −13→13
*T*_min_ = 0.876, *T*_max_ = 1.000	*l* = −13→13
10153 measured reflections	

### Refinement

**Table:**

Refinement on *F*^2^	Primary atom site location: structure-invariant direct methods
Least-squares matrix: full	Secondary atom site location: difference Fourier map
*R*[*F*^2^ > 2σ(*F*^2^)] = 0.049	Hydrogen site location: inferred from neighbouring sites
*wR*(*F*^2^) = 0.133	H-atom parameters constrained
*S* = 1.03	*w* = 1/[σ^2^(*F*_o_^2^) + (0.060*P*)^2^ + 0.0629*P*] where *P* = (*F*_o_^2^ + 2*F*_c_^2^)/3
3813 reflections	(Δ/σ)_max_ = 0.001
220 parameters	Δρ_max_ = 0.21 e Å^−^^3^
0 restraints	Δρ_min_ = −0.20 e Å^−^^3^

### Special details

**Table:**

Experimental. *R*_f_ 0.39 (PE). FT–IR νmax (KBr) cm^-1^: 3057, 3026, 2924, 2849, 1599, 1493, 1450, 1290, 1182, 1080, 1051, 740, 702; ^1^H NMR (400 MHz, CDCl_3_): δ 7.58 (*d*, *J* = 7.2 Hz, 6H), 7.45–7.35 (*m*, 9H), -0.001 (*s*, 9H); ^13^C NMR (100 MHz, CDCl_3_): δ 145.34, 126.63, 125.86, 125.10, 82.71, -0.01; TOF–MS: calculated for C22H24OSiNa 355.1494 [*M* + Na]^+^; found 355.1496. For crystallization 50 mg of compound dissolved in 10 ml mixture of petroleum ether and ethyl acetate (80:20) and left for several days at ambient temperature which yielded white block-shaped crystals.
Geometry. All e.s.d.'s (except the e.s.d. in the dihedral angle between two l.s. planes) are estimated using the full covariance matrix. The cell e.s.d.'s are taken into account individually in the estimation of e.s.d.'s in distances, angles and torsion angles; correlations between e.s.d.'s in cell parameters are only used when they are defined by crystal symmetry. An approximate (isotropic) treatment of cell e.s.d.'s is used for estimating e.s.d.'s involving l.s. planes.
Refinement. Refinement of *F*^2^ against ALL reflections. The weighted *R*-factor *wR* and goodness of fit *S* are based on *F*^2^, conventional *R*-factors *R* are based on *F*, with *F* set to zero for negative *F*^2^. The threshold expression of *F*^2^ > σ(*F*^2^) is used only for calculating *R*-factors(gt) *etc*. and is not relevant to the choice of reflections for refinement. *R*-factors based on *F*^2^ are statistically about twice as large as those based on *F*, and *R*- factors based on ALL data will be even larger.

### Fractional atomic coordinates and isotropic or equivalent isotropic displacement parameters (Å^2^)

**Table:**

	*x*	*y*	*z*	*U*_iso_*/*U*_eq_	
C1	1.0739 (3)	0.7666 (3)	0.4331 (3)	0.0852 (8)	
H1A	1.1326	0.8123	0.4004	0.128*	
H1B	1.1410	0.7283	0.5045	0.128*	
H1C	1.0230	0.8268	0.4730	0.128*	
Si2	0.92918 (7)	0.63614 (6)	0.28129 (6)	0.0539 (2)	
O3	0.82829 (15)	0.68672 (12)	0.14128 (13)	0.0498 (4)	
C4	0.7464 (2)	0.79157 (16)	0.10571 (18)	0.0400 (4)	
C5	0.8614 (2)	0.91551 (18)	0.15089 (18)	0.0432 (4)	
C6	0.8264 (3)	1.0401 (2)	0.1829 (2)	0.0592 (6)	
H6	0.7321	1.0506	0.1827	0.071*	
C7	0.9303 (3)	1.1495 (2)	0.2152 (3)	0.0750 (7)	
H7	0.9054	1.2327	0.2369	0.090*	
C8	1.0695 (3)	1.1357 (3)	0.2155 (2)	0.0757 (7)	
H8	1.1394	1.2092	0.2381	0.091*	
C9	1.1048 (3)	1.0133 (3)	0.1823 (3)	0.0724 (7)	
H9	1.1988	1.0033	0.1819	0.087*	
C10	1.0013 (2)	0.9044 (2)	0.1494 (2)	0.0567 (5)	
H10	1.0263	0.8216	0.1257	0.068*	
C11	0.65606 (19)	0.75217 (17)	−0.05828 (19)	0.0402 (4)	
C12	0.5804 (2)	0.8411 (2)	−0.1289 (2)	0.0524 (5)	
H12	0.5846	0.9253	−0.0765	0.063*	
C13	0.4985 (2)	0.8064 (2)	−0.2761 (2)	0.0641 (6)	
H13	0.4474	0.8670	−0.3216	0.077*	
C14	0.4926 (3)	0.6843 (3)	−0.3546 (2)	0.0700 (7)	
H14	0.4385	0.6612	−0.4535	0.084*	
C15	0.5673 (3)	0.5953 (2)	−0.2860 (2)	0.0722 (7)	
H15	0.5635	0.5115	−0.3390	0.087*	
C16	0.6485 (2)	0.6295 (2)	−0.1386 (2)	0.0575 (6)	
H16	0.6985	0.5682	−0.0937	0.069*	
C17	0.6382 (2)	0.80747 (17)	0.1766 (2)	0.0449 (5)	
C18	0.4855 (3)	0.7525 (2)	0.1031 (2)	0.0642 (6)	
H18	0.4442	0.7104	0.0062	0.077*	
C19	0.3926 (3)	0.7588 (3)	0.1710 (3)	0.0840 (8)	
H19	0.2897	0.7214	0.1194	0.101*	
C20	0.4515 (3)	0.8198 (3)	0.3139 (3)	0.0791 (8)	
H20	0.3888	0.8244	0.3593	0.095*	
C21	0.6020 (3)	0.8737 (2)	0.3888 (3)	0.0720 (7)	
H21	0.6428	0.9135	0.4862	0.086*	
C22	0.6950 (3)	0.8696 (2)	0.3210 (2)	0.0591 (6)	
H22	0.7972	0.9090	0.3731	0.071*	
C23	1.0316 (3)	0.5155 (3)	0.2158 (3)	0.0928 (9)	
H23A	0.9584	0.4491	0.1339	0.139*	
H23B	1.0903	0.4760	0.2897	0.139*	
H23C	1.0992	0.5589	0.1900	0.139*	
C24	0.8034 (3)	0.5569 (3)	0.3385 (3)	0.0996 (9)	
H24A	0.7558	0.6217	0.3772	0.149*	
H24B	0.8634	0.5155	0.4100	0.149*	
H24C	0.7256	0.4923	0.2580	0.149*	

### Atomic displacement parameters (Å^2^)

**Table:**

	*U*^11^	*U*^22^	*U*^33^	*U*^12^	*U*^13^	*U*^23^
C1	0.0620 (16)	0.113 (2)	0.0570 (15)	0.0104 (14)	0.0040 (12)	0.0262 (14)
Si2	0.0457 (3)	0.0637 (4)	0.0548 (4)	0.0159 (3)	0.0170 (3)	0.0306 (3)
O3	0.0494 (8)	0.0512 (8)	0.0441 (8)	0.0196 (6)	0.0133 (6)	0.0184 (6)
C4	0.0384 (10)	0.0422 (10)	0.0390 (10)	0.0125 (8)	0.0151 (8)	0.0139 (8)
C5	0.0404 (10)	0.0526 (12)	0.0327 (10)	0.0080 (8)	0.0122 (8)	0.0131 (8)
C6	0.0608 (14)	0.0518 (13)	0.0635 (14)	0.0057 (10)	0.0294 (12)	0.0108 (10)
C7	0.093 (2)	0.0524 (14)	0.0760 (17)	−0.0047 (13)	0.0414 (15)	0.0074 (12)
C8	0.0711 (17)	0.0803 (18)	0.0635 (16)	−0.0196 (14)	0.0214 (13)	0.0203 (13)
C9	0.0490 (13)	0.098 (2)	0.0768 (17)	0.0027 (13)	0.0263 (12)	0.0402 (15)
C10	0.0492 (12)	0.0700 (14)	0.0577 (13)	0.0128 (10)	0.0249 (10)	0.0277 (11)
C11	0.0327 (9)	0.0467 (11)	0.0417 (10)	0.0085 (8)	0.0168 (8)	0.0128 (9)
C12	0.0490 (12)	0.0538 (12)	0.0482 (12)	0.0118 (9)	0.0145 (10)	0.0167 (10)
C13	0.0542 (13)	0.0818 (17)	0.0516 (13)	0.0179 (11)	0.0126 (11)	0.0309 (12)
C14	0.0564 (14)	0.1006 (19)	0.0398 (12)	0.0143 (13)	0.0126 (10)	0.0129 (13)
C15	0.0705 (16)	0.0758 (16)	0.0510 (14)	0.0195 (13)	0.0190 (12)	−0.0021 (12)
C16	0.0568 (13)	0.0575 (13)	0.0481 (12)	0.0193 (10)	0.0157 (10)	0.0106 (10)
C17	0.0472 (11)	0.0445 (11)	0.0481 (12)	0.0126 (9)	0.0234 (9)	0.0174 (9)
C18	0.0536 (13)	0.0795 (15)	0.0599 (14)	0.0018 (11)	0.0297 (12)	0.0132 (12)
C19	0.0603 (16)	0.109 (2)	0.093 (2)	0.0026 (14)	0.0455 (16)	0.0275 (17)
C20	0.087 (2)	0.0910 (19)	0.095 (2)	0.0259 (16)	0.0664 (18)	0.0376 (16)
C21	0.0926 (19)	0.0823 (17)	0.0612 (15)	0.0316 (15)	0.0482 (15)	0.0258 (13)
C22	0.0595 (13)	0.0661 (14)	0.0517 (13)	0.0145 (11)	0.0258 (11)	0.0139 (11)
C23	0.094 (2)	0.0927 (19)	0.106 (2)	0.0513 (16)	0.0439 (18)	0.0496 (17)
C24	0.096 (2)	0.114 (2)	0.124 (3)	0.0217 (17)	0.059 (2)	0.0756 (19)

### Geometric parameters (Å, °)

**Table:**

C1—Si2	1.849 (2)	C12—H12	0.9300
C1—H1A	0.9600	C13—C14	1.360 (3)
C1—H1B	0.9600	C13—H13	0.9300
C1—H1C	0.9600	C14—C15	1.374 (3)
Si2—O3	1.6379 (14)	C14—H14	0.9300
Si2—C24	1.847 (2)	C15—C16	1.385 (3)
Si2—C23	1.850 (2)	C15—H15	0.9300
O3—C4	1.425 (2)	C16—H16	0.9300
C4—C5	1.535 (2)	C17—C18	1.378 (3)
C4—C17	1.541 (2)	C17—C22	1.389 (3)
C4—C11	1.541 (2)	C18—C19	1.384 (3)
C5—C10	1.382 (3)	C18—H18	0.9300
C5—C6	1.383 (3)	C19—C20	1.370 (4)
C6—C7	1.388 (3)	C19—H19	0.9300
C6—H6	0.9300	C20—C21	1.358 (3)
C7—C8	1.371 (3)	C20—H20	0.9300
C7—H7	0.9300	C21—C22	1.386 (3)
C8—C9	1.365 (3)	C21—H21	0.9300
C8—H8	0.9300	C22—H22	0.9300
C9—C10	1.379 (3)	C23—H23A	0.9600
C9—H9	0.9300	C23—H23B	0.9600
C10—H10	0.9300	C23—H23C	0.9600
C11—C16	1.368 (3)	C24—H24A	0.9600
C11—C12	1.387 (3)	C24—H24B	0.9600
C12—C13	1.385 (3)	C24—H24C	0.9600
			
Si2—C1—H1A	109.5	C14—C13—C12	120.3 (2)
Si2—C1—H1B	109.5	C14—C13—H13	119.9
H1A—C1—H1B	109.5	C12—C13—H13	119.9
Si2—C1—H1C	109.5	C13—C14—C15	119.3 (2)
H1A—C1—H1C	109.5	C13—C14—H14	120.4
H1B—C1—H1C	109.5	C15—C14—H14	120.4
O3—Si2—C24	111.11 (11)	C14—C15—C16	120.5 (2)
O3—Si2—C1	113.53 (10)	C14—C15—H15	119.7
C24—Si2—C1	110.11 (14)	C16—C15—H15	119.7
O3—Si2—C23	103.08 (10)	C11—C16—C15	120.9 (2)
C24—Si2—C23	110.57 (13)	C11—C16—H16	119.6
C1—Si2—C23	108.19 (13)	C15—C16—H16	119.6
C4—O3—Si2	139.79 (11)	C18—C17—C22	117.47 (17)
O3—C4—C5	109.22 (14)	C18—C17—C4	121.84 (17)
O3—C4—C17	108.41 (14)	C22—C17—C4	120.50 (17)
C5—C4—C17	113.56 (14)	C17—C18—C19	121.2 (2)
O3—C4—C11	106.13 (13)	C17—C18—H18	119.4
C5—C4—C11	107.71 (14)	C19—C18—H18	119.4
C17—C4—C11	111.54 (14)	C20—C19—C18	120.3 (2)
C10—C5—C6	117.68 (18)	C20—C19—H19	119.9
C10—C5—C4	119.62 (17)	C18—C19—H19	119.9
C6—C5—C4	122.50 (16)	C21—C20—C19	119.6 (2)
C5—C6—C7	120.7 (2)	C21—C20—H20	120.2
C5—C6—H6	119.6	C19—C20—H20	120.2
C7—C6—H6	119.6	C20—C21—C22	120.4 (2)
C8—C7—C6	120.4 (2)	C20—C21—H21	119.8
C8—C7—H7	119.8	C22—C21—H21	119.8
C6—C7—H7	119.8	C21—C22—C17	121.0 (2)
C9—C8—C7	119.5 (2)	C21—C22—H22	119.5
C9—C8—H8	120.2	C17—C22—H22	119.5
C7—C8—H8	120.2	Si2—C23—H23A	109.5
C8—C9—C10	120.2 (2)	Si2—C23—H23B	109.5
C8—C9—H9	119.9	H23A—C23—H23B	109.5
C10—C9—H9	119.9	Si2—C23—H23C	109.5
C9—C10—C5	121.5 (2)	H23A—C23—H23C	109.5
C9—C10—H10	119.2	H23B—C23—H23C	109.5
C5—C10—H10	119.2	Si2—C24—H24A	109.5
C16—C11—C12	117.98 (18)	Si2—C24—H24B	109.5
C16—C11—C4	121.40 (17)	H24A—C24—H24B	109.5
C12—C11—C4	120.62 (16)	Si2—C24—H24C	109.5
C13—C12—C11	121.0 (2)	H24A—C24—H24C	109.5
C13—C12—H12	119.5	H24B—C24—H24C	109.5
C11—C12—H12	119.5		
			
C24—Si2—O3—C4	78.0 (2)	C5—C4—C11—C12	−54.3 (2)
C1—Si2—O3—C4	−46.8 (2)	C17—C4—C11—C12	71.0 (2)
C23—Si2—O3—C4	−163.56 (18)	C16—C11—C12—C13	0.5 (3)
Si2—O3—C4—C5	74.6 (2)	C4—C11—C12—C13	−179.80 (17)
Si2—O3—C4—C17	−49.6 (2)	C11—C12—C13—C14	−0.8 (3)
Si2—O3—C4—C11	−169.49 (13)	C12—C13—C14—C15	0.6 (3)
O3—C4—C5—C10	31.1 (2)	C13—C14—C15—C16	−0.2 (4)
C17—C4—C5—C10	152.18 (17)	C12—C11—C16—C15	−0.1 (3)
C11—C4—C5—C10	−83.8 (2)	C4—C11—C16—C15	−179.78 (19)
O3—C4—C5—C6	−154.17 (17)	C14—C15—C16—C11	0.0 (3)
C17—C4—C5—C6	−33.0 (2)	O3—C4—C17—C18	−97.1 (2)
C11—C4—C5—C6	91.0 (2)	C5—C4—C17—C18	141.27 (19)
C10—C5—C6—C7	−1.4 (3)	C11—C4—C17—C18	19.3 (2)
C4—C5—C6—C7	−176.2 (2)	O3—C4—C17—C22	77.7 (2)
C5—C6—C7—C8	0.2 (4)	C5—C4—C17—C22	−43.9 (2)
C6—C7—C8—C9	0.6 (4)	C11—C4—C17—C22	−165.84 (18)
C7—C8—C9—C10	−0.3 (4)	C22—C17—C18—C19	−0.1 (3)
C8—C9—C10—C5	−0.9 (4)	C4—C17—C18—C19	174.9 (2)
C6—C5—C10—C9	1.7 (3)	C17—C18—C19—C20	−0.3 (4)
C4—C5—C10—C9	176.73 (19)	C18—C19—C20—C21	−0.3 (4)
O3—C4—C11—C16	8.5 (2)	C19—C20—C21—C22	1.4 (4)
C5—C4—C11—C16	125.35 (18)	C20—C21—C22—C17	−1.8 (3)
C17—C4—C11—C16	−109.40 (19)	C18—C17—C22—C21	1.2 (3)
O3—C4—C11—C12	−171.17 (15)	C4—C17—C22—C21	−173.86 (19)
